# Identification of Shiga-Toxigenic *Escherichia coli* outbreak isolates by a novel data analysis tool after matrix-assisted laser desorption/ionization time-of-flight mass spectrometry

**DOI:** 10.1371/journal.pone.0182962

**Published:** 2017-09-06

**Authors:** Martin Christner, Dirk Dressler, Mark Andrian, Claudia Reule, Orlando Petrini

**Affiliations:** 1 Department of Medical Microbiology, Virology and Hygiene, University Medical Center Hamburg-Eppendorf, Hamburg, Germany; 2 Biotesys GmbH, Esslingen, Germany; 3 ArsNova GmbH, Esslingen, Germany; 4 POLE Pharma Consulting, Breganzona, Switzerland; 5 Swiss Technical Institute of Technology, Zurich, Switzerland; Universidad Nacional de la Plata, ARGENTINA

## Abstract

The fast and reliable characterization of bacterial and fungal pathogens plays an important role in infectious disease control and tracking of outbreak agents. DNA based methods are the gold standard for epidemiological investigations, but they are still comparatively expensive and time-consuming. Matrix-assisted laser desorption/ionization time-of-flight mass spectrometry (MALDI-TOF MS) is a fast, reliable and cost-effective technique now routinely used to identify clinically relevant human pathogens. It has been used for subspecies differentiation and typing, but its use for epidemiological tasks, e. g. for outbreak investigations, is often hampered by the complexity of data analysis. We have analysed publicly available MALDI-TOF mass spectra from a large outbreak of Shiga-Toxigenic *Escherichia coli* in northern Germany using a general purpose software tool for the analysis of complex biological data. The software was challenged with depauperate spectra and reduced learning group sizes to mimic poor spectrum quality and scarcity of reference spectra at the onset of an outbreak. With high quality formic acid extraction spectra, the software’s built in classifier accurately identified outbreak related strains using as few as 10 reference spectra (99.8% sensitivity, 98.0% specificity). Selective variation of processing parameters showed impaired marker peak detection and reduced classification accuracy in samples with high background noise or artificially reduced peak counts. However, the software consistently identified mass signals suitable for a highly reliable marker peak based classification approach (100% sensitivity, 99.5% specificity) even from low quality direct deposition spectra. The study demonstrates that general purpose data analysis tools can effectively be used for the analysis of bacterial mass spectra.

## Introduction

Characterization of bacterial and fungal pathogens is essential in effective infectious disease control and tracking of outbreak agents. DNA based methods such as PCR and sequencing are the gold standard but they are comparatively expensive and time-consuming.

In the last few years, matrix-assisted laser desorption/ionization time-of-flight mass spectrometry (MALDI-TOF MS) fingerprinting has been extensively used to identify clinically relevant human pathogens, including bacteria [1–5], yeasts [6,7] and filamentous fungi [1,8]. Microbial identification is based on the analysis of whole cell mass spectra (mainly representing highly abundant ribosomal proteins) that are compared to reference spectra of well characterized isolates or probed for the presence of known genus- and species-specific mass signals [9]. The technique is fast, reliable, and cost-effective. Critical issues for the further development of MALDI-TOF MS in medical microbiology are the maintenance of reliable reference databases and the identification of discriminatory mass signals to increase phylogenetic resolution within closely related taxa. In recent years, identification algorithms and databases have continuously been expanded and improved by the major suppliers of MALDI-TOF MS based microbial identification systems, but subspecies-level discrimination has mainly been pursued with in-house algorithms and workflows.

The identification of characteristic marker peaks seems to be a promising approach for the rapid subspecies-level classification of important pathogens such as methicillin-resistant *Staphylococcus aureus* [10,11] or typhoid *Salmonella* [12]. During a large outbreak of Shiga-Toxigenic *E*. *coli* (STEC) in northern Germany [13], Christner et al [14] developed a marker peak based MALDI-TOF MS typing scheme by comparing reference spectra of the Shiga-Toxigenic *Escherichia coli* (STEC) outbreak isolate TY-2482 [15] to a random selection of pre-outbreak spectra. Their final classification results were in almost perfect concordance to those obtained by PCR genotyping and multilocus sequence typing (MLST). This study, however, involved the use of complex statistical software and expertise in spectrum analysis to identify the relevant mass signals. A user friendly software solution to detect marker peaks and to classify MALDI-TOF mass spectra for epidemiological purposes would thus be very useful for clinical microbiology laboratories without extensive knowledge in mass spectrometry data analysis.

A.B.O.S. (A Better Omic System; version 1.1.0; Ars Nova AG, Esslingen, Germany) a simple, interactive software for the analysis of omics data has recently been developed to classify and evaluate biological properties or processes in complex datasets. The tool utilises self-learning algorithms that exploit group-specific properties from large datasets and applies a combination of multivariate analysis techniques such as principal component analysis (PCA), weighting the different variables/parameters according to their discriminatory power. Unlike PCA, however, the program can handle data that are not normally distributed and accounts for the presence of outliers and missing data. The software carries out predictive identifications based on pre-assigned learning groups that can be either detected automatically or defined manually. By combining all parameters shared by the members of each learning groups, the software calculates two ideal reference groups and classifies unknown elements based on their relative distance to these groups. Along with the proposed classification of samples, it also identifies the most important parameters that allow differentiating between classes.

The aim of the current study was to investigate the usefulness of this software tool for marker peak detection and classification of MALDI-TOF mass spectra using publicly available data from a large STEC outbreak [14].

## Materials and methods

### Study design

The applicability of the new software for the analysis of bacterial mass spectra was tested with *Escherichia coli* spectra from a large STEC outbreak [14]. To simulate real-world outbreak situations, a limited subset of outbreak and non-outbreak isolates (learning groups) was used to train the software’s built-in classifier and to identify outbreak strain specific mass signals (marker peaks) which could be utilized for marker peak based typing schemes. Performance of the software’s built in classifier was tested by challenging the trained classifier with spectra from the remaining isolates. Performance of marker peak identification was evaluated by the frequency of detection of known outbreak strain marker peaks. All analyses were repeated ten times with different learning group composition. Systematic variation of processing parameters (e. g. spectrum quality and learning group sizes) was employed to test the methods’ robustness. In addition, the software was utilized to identify novel marker peak candidates by analysis of the full dataset.

### Sample spectra

Publicly available MALDI-TOF mass spectra (m/z 3,000 to 20,000) of 294 *Escherichia coli* isolates recovered from various patient samples during a large STEC outbreak were obtained from Dryad [16]. All isolates had been classified as outbreak related (OREC; n = 104) or non-outbreak related *E*. *coli* (NOREC; n = 190) by PCR genotyping and multilocus sequence typing (MLST) [14]. The spectrum collection comprised two complete sets of raw triplicate spectra acquired on a Bruker microflex LT mass spectrometer using direct sample deposition (DSD) or formic acid extraction (FAE). Isolate names started with a 4-digit random number part to facilitate random sampling.

### Spectrum processing

Raw spectra were processed with MALDIquant for R as previously described [14]. After trimming (m/z range 3,000–12,000), smoothing (moving average; half window size: 4), baseline correction (SNIP; half window size: 25) and peak picking (median absolute deviation; half window size: 12; signal to noise ratio (SNR) cut-off: 2, 4, 8, 16, 32), the resulting peak lists were aligned and binned (m/z tolerance: 800 ppm) and exported as intensity matrices (containing information on relative peak intensities) and binary matrices (peak presence/absence) in csv-format. Peak lists from technical replicates were either merged using a ≥2 of 3 threshold after an additional sample-wise alignment/binning step or passed as individual spectra (unmerged).

### A.B.O.S. analysis

Analyses were performed on peak intensity and binary matrices imported from R-generated csv-files. In each session, different subsets of three, five or ten consecutive OREC and NOREC isolates from the randomly ordered dataset were assigned to the respective learning groups to train the built in classifier and to identify discriminatory mass signals. For each input dataset and each tested SNR cut-off and learning group size, ten independent sessions with different learning group composition were conducted.

#### Performance of the built-in classifier

For each session, sensitivity, specificity and accuracy for outbreak strain identification were calculated using the existing reference classification as gold standard. Isolates categorized by A.B.O.S. as indeterminate were counted as NOREC. Measures from ten sessions were averaged to obtain aggregated measures of classification performance.

A.B.O.S. results were compared to a whole spectrum Jaccard distance based classifier implemented with R. Spectra assigned to the OREC learning group in A.B.O.S. were used as outbreak strain reference spectra. For each test strain, the minimum Jaccard distance to the selected outbreak strain reference spectra was determined using the ‘proxy’ package [https://CRAN.R-project.org/package=proxy]. The resulting distance values were subjected to receiver operating characteristic (ROC) analysis using the ‘pROC’ package [https://CRAN.R-project.org/package=pROC].

#### Suitability for marker peak detection

The lists of most significant peaks generated during classification runs were evaluated with respect to presence or absence of signals at m/z 3356, m/z 5442, m/z 6711 and m/z 10884 which are known to represent two outbreak strain maker proteins (NCBI GenPept accession numbers YP_004119749 and WP_000647571 [14]). In addition, the frequency of peak presence in the lists of most significant peaks from ten independent computational runs and a rank score, calculated as Σ_*i*_(11 − *rank in the list of most significant peaks of run*_*i*_), were determined for each peak among the top ten most significant peaks in the analyses with binary matrices from FAE spectra processed with a SNR cut-off of 4.

#### Identification of novel marker peak candidates

For detection of new marker peak candidates, the whole spectrum collection was assigned to A.B.O.S. learning groups according to the reference classification. The top 40 peaks from the resulting list of most significant peaks were further analysed with respect to differences in detection frequencies between OREC and NOREC isolates (disciminatory power) as well as signal intensities (detectability) and detection frequencies among OREC isolates (reproducibility). Pair wise Pearson correlation and Pearson distances between binary signal vectors representing peak presence or absence from NOREC FAE spectra processed with a SNR cut-off of 4 were determined in R to identify correlated peaks. The most promising signals were followed up by manual spectrum inspection and molecular weight matching in publicly accessible protein databases.

## Results

### Classification accuracy

Mean classification accuracy in repeated analyses with learning groups of five isolates and FAE spectra processed with a SNR cut-off of 4 was 98.6% (range: 97.6–99.7%, Table 1). Sensitivity of outbreak strain identification was 100% in nine of 10 test runs at specificities ranging from 96.4 to 99.5% (S1 Table). In all analyses with FAE-spectra, classification results were on or above the receiver operating characteristic curve of the corresponding whole spectrum similarity based classification (S1 Fig).

**Table 1:** Classification accuracy and marker peak detection rates.

Sample prep. ^1^	SNR CUT-OFF^2^	LG size^3^	Peak data^4^	Classification performance [%]^5^			MSP^6^	Marker protein detection frequency [%]^7^				
								MP1		MP2		MP1&2
				Sens.	Spec.	Acc.		p3356	p6711	p5442	p10883	
fae	2	5	QL	99.2	98.4	98.6	39	70	80	50	100	100
	4	3	QL	99.0	93.9	95.6	42	90	50	60	80	80
		5	QL	99.8	98.0	98.6	30	90	100	100	100	100
		10	QL	100	98.1	98.8	24	100	100	100	100	100
	8	5	QL	96.5	98.1	97.6	36	100	100	100	100	100
	16	5	QL	98.7	99.6	99.3	30	70	100	100	100	100
	32	5	QL	91.0	89.5	89.9	29	0	100	30	50	50
	4	5	QN	84.9	95.6	91.7	54	10	10	30	10	10
dsd	4	3	QL	80.7	94.8	89.7	48	0	70	100	90	70
		5	QL	88.3	98.4	94.8	44	0	100	100	100	100
		10	QL	94.0	98.3	96.8	42	0	100	100	100	100

^1^ Method of sample preparation for MALDI-TOF MS measurement: FAE or DSD.

^2^ Signal to noise ratio cut-off used for peak detection.

^3^ Size of learning groups for A.B.O.S. analysis.

^4^ Analysis of qualitative (QL; peak presence or absence) or quantitative (QN; peak intensity) peaklists.

^5^ Mean sensitivity, specificity and accuracy from 10 independent runs.

^6^ Total number of different peaks listed among the top 10 most important peaks in 10 independent A.B.O.S. runs.

^7^ Presence of peaks representing known outbreak strain marker proteins among the ten most significant peaks in 10 independent runs.

### Marker peak detection

Mass signals at m/z 6711 or 3356 and m/z 10884 or 5442, representing singly and doubly charged ions of two previously identified outbreak strain marker proteins (NCBI GenPept accession numbers YP_004119749 and WP_000647571 [14]), were identified as discriminatory peaks in all test runs with FAE spectra processed with a SNR cut-off of 4 and learning groups of five isolates (Table 1). The peaks occupied at least three of the top five positions in the lists of the most relevant mass signals in eight of ten runs (S1 Table).

Only three other signals (at m/z 6601, 6842 and 8801), among the 30 different peaks, that were listed among the ten most significant signals in ten independent A.B.O.S. runs, were found to be relevant in more than 50% of the runs (S1 Table). However, compared to m/z 6711/3356 and m/z 10884/5442, these potential OREC markers showed lower signal intensities and inferior discriminatory power (S2 Table). Consequently, m/z 6842 had already been rejected as suitable marker peak in a previous study [14].

**Fig 1:**
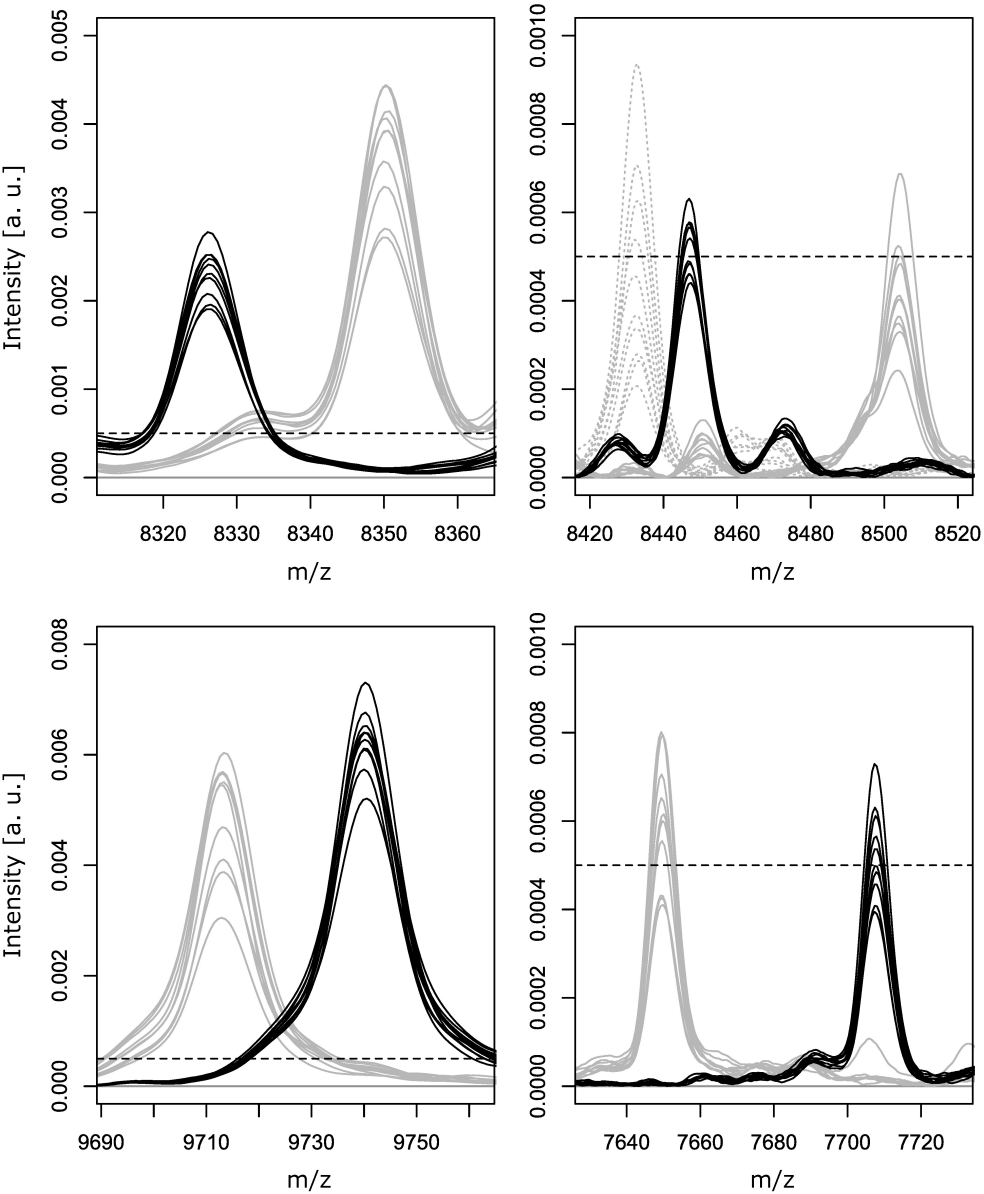
NOREC marker peaks detected by A.B.O.S. analysis.

A.B.O.S. analysis of the fully classified dataset identified twelve additional peaks with satisfactory signal intensities (>0.0005) and detection frequency differences (> 30%) between OREC and NOREC strains (highlighted in S2 Table). Seven potential OREC markers at m/z 3446, 4164, 4871, 5872, 7708, 8326 and 9740 were found to be of little additional value due to their comparably high detection frequencies among NOREC isolates (0.48–0.69, S2 Table). In contrast, two pairs of correlated signals at m/z 8350 and 4176 and m/z 9713 and 4857, turned out to be fairly prevalent NOREC markers (detected in 32% and 31% of NOREC spectra) which could complement OREC specific signals in marker peak based typing schemes (S2 Table). Distribution of markers 8350/4176 and 9713/4857 among NOREC isolates was found to be in almost perfect inverse correlation to OREC markers 8326/4164 (Pearson distance D = 0.33, Pearson correlation coefficient r = -0.90) and 9740/4871 (D = 0.32, r = -0.93). Assuming peaks with inverse distribution to represent different variants of the same protein, additional NOREC markers at m/z 7649/3825, 8433/4217 and 8505/4253 were identified via their low Pearson distance to OREC markers 7708/3854 (D = 0.39, r = -0.76) and 8447/4224 (D = 0.31, r = -0.94), respectively. In all these cases, signal exclusivity of negatively correlated peaks could be confirmed by manual spectrum inspection. Detection frequencies of these markers among NOREC isolates were 24% (7649/3825) and 32% (8433/4217 or 8505/4253). Presence of at least one of these NOREC markers correctly classified 96 of 190 (51%) NOREC study isolates as not outbreak related with no false positives among 140 OREC isolates (Fig 1).

The peak at m/z 4777.8 was discarded as marker candidate because of inconsistent detectability among OREC isolates (53%). From the 21 remaining low intensity signals, 13 were classified as truly discriminatory (OREC markers: m/z 3854, 4224, 4402, 8282, 8473, 9801, 10464; NOREC markers: m/z 4407, 6669, 7909, 7926, 8814, 11710) based on manual spectrum inspection and analysis of overall peak frequency distribution (S2 Table, S2 Fig). The remaining signals (m/z 3086, 3207, 4756, 5589, 5899, 8119, 9209, 10922) were deemed non-informative.

### Effect of learning group sizes, spectrum quality and processing parameters

Increasing learning group sizes resulted in improved sensitivity of OREC detection and improved classification accuracy (Table 1). Signals representing the two proteins required for reliable marker peak based outbreak strain identification were detected in all analysis sessions with learning group sizes of five and ten from FAE and DSD spectra processed with signal to noise ratio cut-off 4. Even learning groups of only three isolates resulted in correct marker peak identification in the majority of cases and satisfactory classification of FAE spectra.

As expected from other studies with marker peak based or whole spectrum similarity based classification approaches (5), A.B.O.S. analysis yielded better results with FAE than with DSD samples. The effect was more pronounced with smaller learning group sizes. Sensitivity for outbreak strain detection was 6.0%, 11.5% and 18.3% higher with FAE samples using learning group sizes of ten, five and three isolates, respectively (Table 1).

Performance was influenced to a lesser extent by the chosen SNR cut-off. Classification accuracy with FAE samples and learning group size five was optimal at SNR 4 (99.8% sensitivity; 98.0% specificity). Sensitivity, however, never dropped below 95% for SNR cut-offs between 2 and 16 (Table 1). Only highly depauperated peak lists, produced with a SNR cut-off of 32 (34.5 peaks per sample, S3 Table), led to a marked decrease in classification accuracy (91.0% sensitivity; 89.5% specificity) and marker peak detection frequency (50%).

The use of intensity matrices instead of binary matrices for A.B.O.S. analysis did not improve classification results. On the contrary, marker peak detection frequency and measures of classification accuracy were markedly reduced (Table 1).

No systematic differences were observed between different strategies for the handling of technical replicates (merging vs. analysis as individual spectra; data not shown).

## Discussion and conclusions

When applied to high quality replicate MALDI-TOF mass spectra from a large STEC outbreak, the A.B.O.S. software’s built in classifier successfully distinguished outbreak related from non-outbreak related isolates based on the analysis of as few as 10 reference spectra. The software thereby outperformed a previously evaluated whole spectrum Jaccard-distance based classifier [14]. In our sample population (prevalence = 0.35), A.B.O.S. classification resulted in acceptable positive and negative predictive values (96.5% PPV, 99.9% NPV). At lower prevalence however, the observed specificity would require additional confirmatory tests to rule in outbreak isolates (PPV of 84.6%, 72.3% and 33.3% at prevalence 0.1, 0.05 and 0.01). Performance was markedly reduced when the classifier was challenged with lower quality DSD spectra.

These findings fit our previous experience with reference spectrum based subspecies differentiation [14]. Besides the limited intra-species variability of whole cell microbial MALDI-TOF mass spectra, supervised machine learning algorithms are challenged by the substantial inter-assay variability of peaklists derived from these spectra. In our sample collection, only a handful of the more than 100 peaks of a typical *E*. *coli* mass spectrum was relevant for outbreak strain identification. Among those, only signals representing two previously identified outbreak strain marker proteins could be detected in 100% of outbreak strain spectra, even when using high quality FAE replicate spectra. Training on limited data sets is thus prone to producing overfitted models giving too much weight to mass signals with unsatisfactory discriminatory power. In our study, this problem was illustrated by the effect of varying spectrum processing and analysis parameters. Incorporation of peak intensity data, which is known to suffer from high inter-assay variability, inflated the number of supposedly discriminatory peaks up to the point where highly specific marker protein peaks where no longer reliably recognized as relevant for classification. In contrast, increasing SNR cut-offs were accompanied by reduced variability in the list of discriminatory peaks and more reliable detection of known outbreak strain marker peaks. As expected, larger learning groups could only partly overcome these general limitations: increasing learning group size beyond ten isolates did not lead to further improvements of classification accuracy.

In a previous study of the investigated dataset, outbreak strain identification based on a limited set of carefully selected marker peaks outperformed all tested spectrum similarity based classifiers and provided high classification accuracy from FAE and DSD spectra [14]. A.B.O.S.’s list of most significant peaks, generated with each analysis, could provide candidate signals for such marker peak based typing schemes. In our repeated analyses, the crucial peaks at m/z 6711 and m/z 10833 were reliably recognized over a wide range of sample processing and analysis parameters. Currently, however, the software lacks the capability for manual weighting of discriminatory signals, which would be required to set up and perform strict marker peak based classification.

While the current study did not identify useful additional outbreak strain marker peaks, our analysis revealed four unrecognized proteins (represented by at least 10 different mass signals) with sufficient variability among endemic *E*. *coli* to complement classification with known OREC specific markers. Notably, implementation of these signals (particularly the presence of m/z 8433 and absence of m/z 8447) would have prevented the only misclassification observed with the aforementioned marker peak based typing scheme.

In contrast to the R code employed by Christner et al. [14], the interactive A.B.O.S. sessions could be performed without expert knowledge in mass spectrometry or analysis of complex datasets. The software provided rapid insight into our complex dataset and produced satisfactory results from a minimal number of reference spectra. Unfortunately, A.B.O.S. currently lacks modules for spectrum processing and visualization, which would be essential for a standalone use in MALDI-TOF MS data analysis.

Applicability of our approach to other species and outbreak scenarios heavily depends on the general MALDI-TOF MS ‘typeability’ of the respective species (i. e. the intra-species spectral variability) and the amount and quality of information available for the validation of marker peak candidates. Without comprehensive data on the proteins represented in mass spectra of epidemiologically relevant species or representative spectrum databases to reliably predict peak frequencies in the target population, quick and inexpensive MALDI-TOF MS based classification approaches might still be useful to stratify isolates for second tier testing.

In conclusion, the study demonstrates the successful application of a general-purpose data analysis tool for the analysis of bacterial mass spectra from a large STEC outbreak. The ease of use and interactive nature of such tools might encourage microbiologists to explore more comprehensively the considerable amounts of spectrum data routinely acquired in many laboratories.

## Supporting information

S1 Table A.B.O.S. classification results (FAE samples, SNR cut-off 4, learning group size 5). 1 Boldface peaks represent previously identified outbreak strain marker proteins [Christner et al.; PLoS ONE 2014;9(7):e101924].
(DOCX)

S2 Table Characteristics of discriminatory peaks identified by A.B.O.S. analysis of the complete spectrum dataset (FAE spectra, SNR cut-off 4). 1 Peak frequency among OREC isolates minus peak frequency among NOREC isolates 2 Rankscore indicates frequency of peak presence and list position in the lists of most significant peaks from ten independent A.B.O.S. runs with learning group size of 10, 5 and 3 (see methods section for details). Cell shading indicates signals with mean intensity ≥ 0.0005 and detection frequency differences ≥ 0.3 or ≤ -0.3.
(DOCX)

S3 Table Number of peaks in peaklists from FAE and DSD spectra processed with varying SNR cut-offs. (DOCX)

S1 Fig A.B.O.S. classification results (points) and ROC-curves from Jaccard-distance based classification using ten different sets of reference strains (SNR cut-off 4, learning group size 5). FAE spectra: panels 1 through 1; DSD spectra: panels 11 through 20.
(DOCX)

S2 Fig Spectra from twenty OREC (red) and forty NOREC (grey) isolates at peak positions listed in S2 Table. The grey dashed lines indicate relative signal intensity of 0.0005.
(DOCX)
