# Supplementary material for: Identification of Shiga-Toxigenic Escherichia coli outbreak isolates by a novel data analysis tool after matrix-assisted laser desorption/ionization time-of-flight mass spectrometry
Source: PLoS One. 2017 Sep 6;12(9):e0182962. doi: 10.1371/journal.pone.0182962 (PMC5587271; doi:10.1371/journal.pone.0182962)
Supplement: S1 Table — 1 Boldface peaks represent previously identified outbreak strain marker proteins [Christner et al.; PLoS ONE 2014;9(7):e101924]. (DOCX) [file pone.0182962.s001.docx]

S1 Table. A.B.O.S. classification results (FAE samples, SNR cut-off 4, learning group size 5).

| **ABOS run** | **OREC learning group** | **NOREC learning group** | **Sens.** | **Spec.** | **Most important peaks^1^** |
| --- | --- | --- | --- | --- | --- |
| 1 | 10182819; 10402733; 10624672; 10982689; 12912692 | 10263180; 10303688; 10578818; 10666319; 10853120 | 1.00 | 0.98 | **3356**; **5442**; **6711**; **10884**; 3086; 6601; 6842; 8801; 8814; 11710 |
| 2 | 18624809; 18994751; 19214755; 19233549; 20133751 | 17518453; 18693142; 18745321; 18873883; 19503882 | 1.00 | 0.98 | **3356**; 3446; **10883**; **5442**; 6601; **6711**; 6842; 8801; 3179; 4402 |
| 3 | 24533920; 24645048; 24732694; 25534031; 25652709 | 27793129; 27973126; 28183897; 28473900; 28964700 | 0.98 | 0.97 | 6842; **3356**; **5442**; 6601; **6711**; **10883**; 5899; 3086; 3207; 3446 |
| 4 | 32052720; 32304674; 32394707; 32833854; 33043647 | 34823119; 35005542; 35153587; 35883750; 35915128 | 1.00 | 0.98 | **5442**; 8801; **10883**; 4407; 4684; 4778; **6711**; 6842; 8473; 8814 |
| 5 | 39043672; 41624055; 42633909; 43474761; 43513799 | 40649696; 40939128; 41777872; 41783426; 42149745 | 1.00 | 0.98 | **6711**; **10883**; **3356**; **5442**; 6842; 8801; 9801; 3854; 4510; 4756 |
| 6 | 51444012; 52924646; 53272696; 55324053; 55773965 | 48147786; 48383116; 48653866; 48866877; 48935079 | 1.00 | 0.99 | 8801; **3356**; **6711**; 8814; **10883**; 4224; 4756; **5442**; 6601; 6734 |
| 7 | 62694789; 64553721; 65812777; 66035067; 66054186 | 59619215; 59807647; 59943122; 60027445; 60225213 | 1.00 | 0.98 | **5442**; **10883**; **6711**; 6842; 8801; 3207; **3356**; 4402; 4756; 6601 |
| 8 | 70064635; 70093852; 70933332; 71934740; 73383879 | 68296598; 68893157; 68953102; 70929740; 71179423 | 1.00 | 0.96 | **3356**; **5442**; **6711**; 6842; 8473; **10883**; 3854; 4224; 8801; 3350 |
| 9 | 76724157; 77174093; 78244112; 79024788; 79224123 | 78714069; 80833136; 81163480; 82062803; 82863121 | 1.00 | 0.96 | 4756; **6711**; 8801; **10883**; **3356**; **5442**; 6601; 7926; 8119; 8814 |
| 10 | 83933990; 87302721; 89182724; 89542714; 92932693 | 91763130; 92083793; 92803871; 92879116; 93203443 | 1.00 | 0.98 | **5442**; 6601; 8801; 10653; **10883**; **3356**; 3854; 5589; **6711**; 3446 |

^1^ Boldface peaks represent previously identified outbreak strain marker proteins [Christner et al.; PLoS ONE 2014;9(7):e101924].
