# Supplementary material for: Identification of Shiga-Toxigenic Escherichia coli outbreak isolates by a novel data analysis tool after matrix-assisted laser desorption/ionization time-of-flight mass spectrometry
Source: PLoS One. 2017 Sep 6;12(9):e0182962. doi: 10.1371/journal.pone.0182962 (PMC5587271; doi:10.1371/journal.pone.0182962)
Supplement: S2 Table — 1 Peak frequency among OREC isolates minus peak frequency among NOREC isolates 2 Rankscore indicates frequency of peak presence and list position in the lists of most significant peaks from ten independent A.B.O.S. runs with learning group size of 10, 5 and 3 (see methods section for details). Cell shading indicates signals with mean intensity ≥ 0.0005 and detection frequency differences ≥ 0.3 or ≤ -0.3. (DOCX) [file pone.0182962.s002.docx]

S2 Table. Characteristics of discriminatory peaks identified by A.B.O.S. analysis of the complete spectrum dataset (FAE spectra, SNR cut-off 4).

| **m/z** | **Freqdiff.^1^** | **Mean int.** | **Freq. OREC** | **Mean int. OREC** | **Freq. NOREC** | **Mean int. NOREC** | **Rankscore (Freq.) Lg10^2^** | **Rankscore (Freq.) Lg5^2^** | **Rankscore (Freq.) Lg3^2^** |
| --- | --- | --- | --- | --- | --- | --- | --- | --- | --- |
| 10883.4 | 0.96 | 0.00069 | 1.00 | 0.00069 | 0.04 | 0.00059 | 85 (1.0) | 70 (1.0) | 37 (0.8) |
| 6710.9 | 0.93 | 0.00176 | 1.00 | 0.00176 | 0.07 | 0.00089 | 83 (1.0) | 68 (1.0) | 33 (0.5) |
| 3356.3 | 0.87 | 0.00035 | 1.00 | 0.00035 | 0.13 | 0.00018 | 72 (1.0) | 71 (0.9) | 61 (0.9) |
| 5442.2 | 0.86 | 0.00058 | 1.00 | 0.00058 | 0.14 | 0.00024 | 69 (1.0) | 78 (1.0) | 55 (0.6) |
| 8800.7 | 0.82 | 0.00013 | 0.98 | 0.00013 | 0.16 | 0.00011 | 59 (1.0) | 54 (0.9) | 27 (0.5) |
| 6842.0 | 0.80 | 0.00020 | 0.87 | 0.00020 | 0.06 | 0.00013 | 50 (0.9) | 41 (0.7) | 32 (0.5) |
| 6600.7 | 0.69 | 0.00013 | 0.96 | 0.00013 | 0.27 | 0.00013 | 37 (0.9) | 34 (0.7) | 30 (0.4) |
| 3445.5 | 0.51 | 0.00062 | 0.99 | 0.00037 | 0.48 | 0.00062 | 15 (0.4) | 11 (0.3) | 13 (0.3) |
| 3854.4 | 0.48 | 0.00015 | 0.94 | 0.00013 | 0.46 | 0.00015 | 6 (0.2) | 11 (0.3) | 43 (0.8) |
| 8473.1 | 0.47 | 0.00011 | 0.97 | 0.00011 | 0.50 | 0.00011 | 8 (0.3) | 8 (0.2) | 11 (0.2) |
| 8814.4 | -0.47 | 0.00012 | 0.00 | - | 0.47 | 0.00012 | 4 (0.2) | 11 (0.4) | 9 (0.1) |
| 8119.1 | -0.47 | 0.00010 | 0.05 | 0.00009 | 0.52 | 0.00010 | 9 (0.3) | 2 (0.1) | - |
| 7925.8 | -0.44 | 0.00013 | 0.12 | 0.00011 | 0.56 | 0.00013 | 7 (0.3) | 3 (0.1) | 6 (0.1) |
| 4755.6 | 0.41 | 0.00029 | 0.81 | 0.00014 | 0.40 | 0.00029 | - | 17 (0.4) | - |
| 4224.3 | 0.41 | 0.00015 | 0.99 | 0.00014 | 0.58 | 0.00015 | 5 (0.1) | 8 (0.2) | 16 (0.4) |
| 8282.0 | 0.40 | 0.00015 | 0.98 | 0.00014 | 0.58 | 0.00015 | - | - | - |
| 4407.4 | -0.39 | 0.00011 | 0.00 | - | 0.39 | 0.00011 | 4 (0.1) | 7 (0.1) | 8 (0.2) |
| 9800.9 | 0.39 | 0.00015 | 0.64 | 0.00015 | 0.25 | 0.00013 | 5 (0.1) | 4 (0.1) | - |
| 10922.0 | 0.39 | 0.00014 | 0.96 | 0.00014 | 0.57 | 0.00011 | - | - | - |
| 5899.4 | 0.38 | 0.00037 | 0.76 | 0.00015 | 0.38 | 0.00037 | 11 (0.3) | 4 (0.1) | - |
| 5872.1 | 0.34 | 0.00154 | 1.00 | 0.00093 | 0.66 | 0.00154 | - | - | - |
| 7908.9 | -0.33 | 0.00025 | 0.00 | - | 0.33 | 0.00025 | - | - | - |
| 8350.2 | -0.33 | 0.00354 | 0.00 | - | 0.33 | 0.00354 | - | - | - |
| 11710.4 | -0.33 | 0.00015 | 0.00 | - | 0.33 | 0.00015 | - | 1 (0.1) | 4 (0.1) |
| 4870.5 | 0.32 | 0.00288 | 0.99 | 0.00288 | 0.67 | 0.00227 | 3 (0.1) | - | - |
| 4777.8 | -0.32 | 0.00090 | 0.53 | 0.00062 | 0.85 | 0.00090 | 1 (0.1) | 5 (0.1) | 4 (0.2) |
| 9740.3 | 0.32 | 0.00637 | 0.99 | 0.00637 | 0.67 | 0.00520 | - | - | - |
| 4163.8 | 0.32 | 0.00116 | 1.00 | 0.00116 | 0.68 | 0.00107 | - | - | 3 (0.1) |
| 7707.7 | 0.32 | 0.00052 | 1.00 | 0.00052 | 0.68 | 0.00051 | - | - | - |
| 8326.2 | 0.31 | 0.00228 | 1.00 | 0.00228 | 0.69 | 0.00214 | - | - | - |
| 4175.7 | -0.31 | 0.00188 | 0.00 | - | 0.31 | 0.00188 | - | - | 8 (0.2) |
| 4401.5 | 0.30 | 0.00011 | 0.37 | 0.00010 | 0.06 | 0.00011 | 4 (0.2) | 4 (0.2) | 6 (0.2) |
| 4856.9 | -0.30 | 0.00229 | 0.00 | - | 0.30 | 0.00229 | - | - | - |
| 9713.4 | -0.30 | 0.00537 | 0.00 | - | 0.30 | 0.00537 | - | - | - |
| 6668.8 | -0.29 | 0.00014 | 0.00 | - | 0.29 | 0.00014 | - | - | - |
| 3206.7 | -0.29 | 0.00011 | 0.13 | 0.00010 | 0.43 | 0.00011 | - | 7 (0.2) | 7 (0.2) |
| 10464.3 | 0.29 | 0.00133 | 1.00 | 0.00101 | 0.71 | 0.00133 | - | - | - |
| 3086.0 | 0.29 | 0.00022 | 0.56 | 0.00014 | 0.27 | 0.00022 | 2 (0.1) | 9 (0.2) | 4 (0.2) |
| 9209.4 | 0.29 | 0.00018 | 0.89 | 0.00016 | 0.61 | 0.00018 | - | - | 8 (0.1) |
| 5589.2 | 0.29 | 0.00013 | 0.38 | 0.00013 | 0.09 | 0.00011 | 7 (0.2) | 3 (0.1) | 9 (0.1) |

^1^ Peak frequency among OREC isolates minus peak frequency among NOREC isolates
^2^ Rankscore indicates frequency of peak presence and list position in the lists of most significant peaks from ten independent A.B.O.S. runs with learning group size of 10, 5 and 3 (see methods section for details).
Cell shading indicates signals with mean intensity ≥ 0.0005 and detection frequency differences ≥ 0.3 or ≤ -0.3.
