# Supplementary material for: Identification of Shiga-Toxigenic Escherichia coli outbreak isolates by a novel data analysis tool after matrix-assisted laser desorption/ionization time-of-flight mass spectrometry
Source: PLoS One. 2017 Sep 6;12(9):e0182962. doi: 10.1371/journal.pone.0182962 (PMC5587271; doi:10.1371/journal.pone.0182962)
Supplement: S3 Table — (DOCX) [file pone.0182962.s003.docx]

S3 Table: Number of peaks in peaklists from FAE and DSD spectra processed with varying SNR cut-offs.

| **SNR cut-off** | **FAE** | | | **DSD** | | |
| --- | --- | --- | --- | --- | --- | --- |
|  | **Mean** | **Max** | **Min** | **Mean** | **Max** | **Min** |
| 2 | 152.0 | 235 | 112 | 161.8 | 247 | 113 |
| 4 | 93.3 | 121 | 78 | 94.8 | 124 | 77 |
| 8 | 67. 8 | 80 | 51 | 64.1 | 80 | 49 |
| 12 | 56.9 | 68 | 43 | 53,0 | 66 | 38 |
| 16 | 50.5 | 61 | 38 | 47.0 | 58 | 35 |
| 20 | 45.7 | 56 | 31 | 43.1 | 54 | 32 |
| 24 | 41.9 | 51 | 28 | 40.4 | 50 | 25 |
| 32 | 34.5 | 45 | 23 | 36.3 | 45 | 21 |
