# Supplementary material for: Identification of Shiga-Toxigenic Escherichia coli outbreak isolates by a novel data analysis tool after matrix-assisted laser desorption/ionization time-of-flight mass spectrometry
Source: PLoS One. 2017 Sep 6;12(9):e0182962. doi: 10.1371/journal.pone.0182962 (PMC5587271; doi:10.1371/journal.pone.0182962)
Supplement: S1 Fig — FAE spectra: panels 1 through 1; DSD spectra: panels 11 through 20. (DOCX) [file pone.0182962.s004.docx]

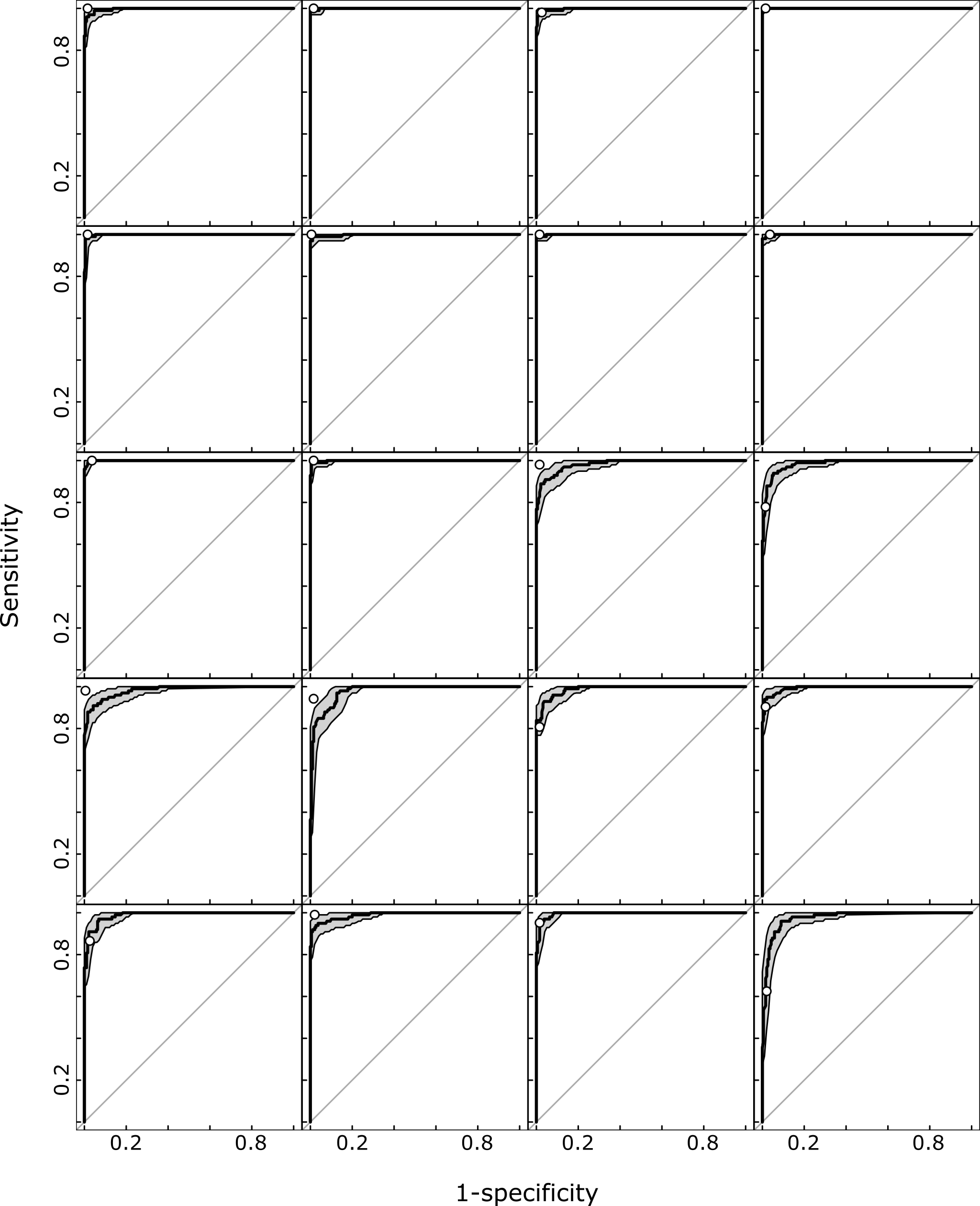


S1 Fig. A.B.O.S. classification results (points) and ROC-curves from Jaccard-distance based classification using ten different sets of reference strains (SNR cut-off 4, learning group size 5).
FAE spectra: panels 1 through 10; DSD spectra: panels 11 through 20.
