# Supplementary material for: Identification of Shiga-Toxigenic Escherichia coli outbreak isolates by a novel data analysis tool after matrix-assisted laser desorption/ionization time-of-flight mass spectrometry
Source: PLoS One. 2017 Sep 6;12(9):e0182962. doi: 10.1371/journal.pone.0182962 (PMC5587271; doi:10.1371/journal.pone.0182962)
Supplement: S2 Fig — The grey dashed lines indicate relative signal intensity of 0.0005. (DOCX) [file pone.0182962.s005.docx]

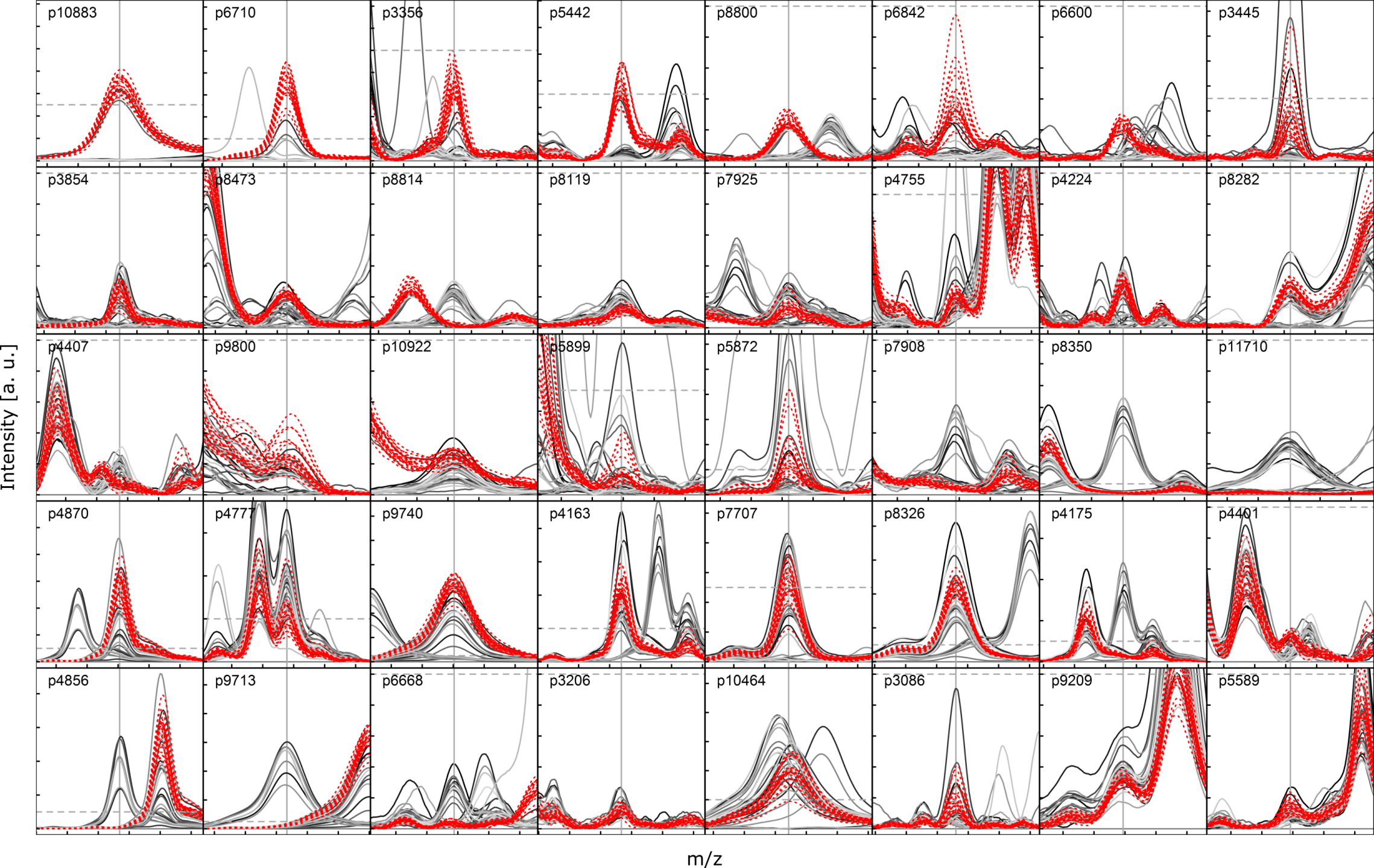


S2 Fig. Spectra from twenty OREC (red) and forty NOREC (grey) isolates at peak positions listed in S2 Table. The grey dashed lines indicate relative signal intensity of 0.0005.
